# Integration of Endocuff‐Assisted and Computer‐Aided Colonoscopy: A Meta‐Analysis of Randomized Controlled Trials

**DOI:** 10.1002/jgh3.70356

**Published:** 2026-02-14

**Authors:** Umar Akram, Eeshal Fatima, Shahzaib Ahmed, Eeman Ahmad, Hareesha Rishab Bharadwaj, Muhammad Hassan Ahmad, Muhammad Sohaib, Khabab Abbasher Hussien Mohamed Ahmed, Dushyant Singh Dahiya

**Affiliations:** ^1^ Department of Medicine Allama Iqbal Medical College Lahore Pakistan; ^2^ Department of Medicine Services Institute of Medical Sciences Lahore Pakistan; ^3^ Department of Medicine Fatima Memorial Hospital College of Medicine and Dentistry Lahore Pakistan; ^4^ Faculty of Biology Medicine and Health The University of Manchester Manchester UK; ^5^ UC Health Parkview Medical Center Pueblo Colorado USA; ^6^ Faculty of Medicine University of Khartoum Khartoum Sudan; ^7^ Department of Gastroenterology, Hepatology and Nutrition The University of Kansas Kansas USA

## Abstract

**Introduction:**

Colorectal cancer (CRC) is a leading cause of cancer‐related deaths, with missed lesions during colonoscopy contributing to increased mortality. AI‐assisted computer‐aided detection (CADe) systems help reduce adenoma miss rates, and their integration with mucosal exposure devices like EndoCuff vision may enhance detection. This meta‐analysis assesses the effectiveness of combining CADe and EndoCuff vision‐assisted colonoscopy (EAC) compared to standard colonoscopy or CADe alone.

**Methods:**

A comprehensive literature search was conducted in MEDLINE, Embase, and clinicaltrials.gov up to November 2024. Only randomized controlled trials (RCTs) comparing CADe+EAC with CADe alone or standard colonoscopy and reporting adenoma detection rate (ADR) were included. Statistical analysis was performed using R version 4.4.0, with mean differences (MDs) and risk ratios (RRs) reported with 95% confidence intervals (CIs).

**Results:**

Four RCTs with 3333 participants were included. Among them, 1228 underwent CADe+EAC, while 554 received standard colonoscopy and 1236 underwent CADe alone. CADe+EAC significantly improved ADR (RR: 1.36; 95% CI: 1.19–1.56), advanced ADR (RR: 1.62; 95% CI: 1.16–2.25), sessile serrated lesion detection rate (RR: 1.95; 95% CI: 1.46–2.61), and mean adenomas per colonoscopy (APC) (MD: 0.50; 95% CI: 0.49–0.52) compared to standard colonoscopy. Compared to CADe alone, CADe+EAC further improved ADR (RR: 1.13; 95% CI: 1.04–1.22) and mean APC (MD: 0.15; 95% CI: 0.07–0.24). However, withdrawal and insertion times were significantly lower in the CADe+EAC group.

**Conclusion:**

The combination of CADe and EAC enhances adenoma detection compared to standard colonoscopy and CADe alone. Further multi‐center trials with diverse AI models are needed to confirm its effectiveness.

## 1 Introduction

Colorectal cancer (CRC) is the third most commonly diagnosed cancer globally, and the second most common cause of cancer‐related deaths [1]. A significant factor contributing to rising morbidity and mortality of CRC is overlooked lesions during colonoscopy, which can result from recognition failure (missing visible lesions on the screen) or incomplete mucosal exposure due to the complexity of colorectal anatomy or suboptimal withdrawal methods [2, 3, 4, 5]. To address this, several techniques like image‐enhanced endoscopy, fold‐flattening devices, wide‐field colonoscopes, and second examinations of the right colon have been implemented [6, 7, 8, 9]. While these approaches can help detect lesions hidden in blind spots, their effectiveness in addressing detection failures by endoscopists remains uncertain.

In recent years, artificial intelligence (AI)–assisted colonoscopy and the subsequent formation of computer‐aided detection (CADe) systems have gained significant clinical interest, as it is expected to reduce the adenoma miss rate by minimizing human error in polyp detection. CADe systems are developed on deep convolutional neural network models [10, 11, 12], and, according to a meta‐analysis of 12 studies, have been found to cause a significant increase in adenoma detection rate (ADR) when compared to non‐CADe systems [13].

Lately, these CADe systems have been integrated with mucosal exposure devices like EndoCuff Vision, significantly enhancing detection rates. Clinical trials have consistently shown that combining CADe with such devices enhances lesion detection accuracy, reduces the adenoma miss rate, and ultimately improves colonoscopy outcomes. While several meta‐analyses have been conducted on the efficacy of colonoscopy with AI‐assisted CADe systems, EndoCuff vision‐assisted colonoscopy (EAC), and the comparison of both modalities, none have been conducted on the efficacy of combining both. The aim of this meta‐analysis is to pool presently available data on the combination of CADe and EAC and to assess its effectiveness in comparison to standard colonoscopy or CADe alone.

## 2 Methods

This systematic review and meta‐analysis was conducted according to the guidelines provided by the Cochrane Handbook for Systematic Reviews of Interventions and Preferred Reporting Items for Systematic Reviews and Meta‐Analysis (PRISMA) [14]. This review was registered with PROSPERO, CRD42024625411. Ethical approval was not required because the study utilized already published publicly available data.

### 2.1 Data Sources and Search Strategy

We searched three electronic databases—MEDLINE (via PubMed), EMBASE (via Ovid), and Cochrane CENTRAL Library—from inception till December 2024. The clinical trial registration database (clinicaltrials.gov) was also searched for relevant trials. Reference lists in review articles identified during this search and the final included articles were checked to identify additional potentially eligible studies. Detailed search strategies are summarized in Table S1.

### 2.2 Study Selection

Two investigators independently assessed all potentially relevant studies. Only randomized controlled trials (RCTs) comparing EAC in combination with CADe (CADe+EAC) with either standard colonoscopy or CADe alone were considered eligible. Only studies published in the English language were considered. Review articles, commentaries, conference abstracts, studies lacking sufficient data, animal studies, and non‐randomized trials were excluded. Any discrepancy between the two investigators was resolved by consulting a third investigator. Studies including patients undergoing endoscopy for dysplasia assessment in inflammatory bowel disease, surveillance of previous cancer, or surveillance for hereditary polyposis syndromes, and studies using video reviews rather than real‐time CADe systems were excluded.

### 2.3 Data Extraction

Two investigators independently extracted the following baseline data from finalized studies: first author's surname, year of publication, study location, study type (single‐center or multi‐center), study design, Intervention and control details, number of participants, mean age of participants, mean Boston bowel preparation scale (BBPS) score, procedure setting, type of computer‐aided system used, and endoscopist experience. Data for the following clinical outcomes was extracted: ADR, advanced ADR (AADR), sessile serrated lesion detection rate (SSLDR), mean adenomas per colonoscopy (mean APC), mean withdrawal time, and mean insertion time. Any discrepancy between the two investigators was resolved by consulting a third investigator.

### 2.4 Quality Assessment

Two reviewers independently used version 2 of the Cochrane risk‐of‐bias tool for randomized trials (RoB 2) to assess the quality of the included studies. This tool evaluates five domains of a randomized study to reach an overall risk of bias judgment: bias in the randomization process, bias due to deviations from the intended interventions, bias due to missing outcome data, bias in the measurement of the outcome, and bias due to selective reporting of results. Disagreements were resolved by a third investigator.

### 2.5 Statistical Analysis

The statistical analysis was conducted on R version 4.4.0 using “meta” and “metasens” packages. For continuous outcomes mean differences (MDs) with 95% confidence intervals (CIs) were calculated. For dichotomous outcomes risk ratios (RRs) with 95% CIs were calculated. The RRs with 95% CIs were pooled using the Mantel–Haenszel method in a random effects model [15]. The MDs were pooled using the inverse variance method. The variance was calculated using the Paule‐Mandel estimator and the restricted maximum likelihood estimator for dichotomous and continuous outcomes respectively [16, 17]. Heterogeneity was assessed using the cutoff values in accordance with the Cochrane Handbook of Systematic Reviews of Interventions for the Higgins *I* ^2^ statistic, keeping in view the results of the Chi^2^ test—0% to 40%: low heterogeneity; 30% to 60%: moderate heterogeneity; 50% to 90%: substantial heterogeneity; and 75% to 100%: considerable heterogeneity [18]. Publication bias cannot be assessed as the number of studies are less than 10. Wherever more than two studies were included, we conducted a sensitivity analysis by omitting one study at a time. A two‐tailed *p* value < 0.05 was considered statistically significant in all instances.

### 2.6 Certainty of Evidence

We used the Grading of Recommendations Development, Assessment and Evaluation (GRADE) approach to determine the certainty of evidence [19]. The summary of effects table was generated via GRADEpro Guideline Development Tool (Table 4) [20].

## 3 Results

### 3.1 Characteristics of Included Studies

**FIGURE 1:**
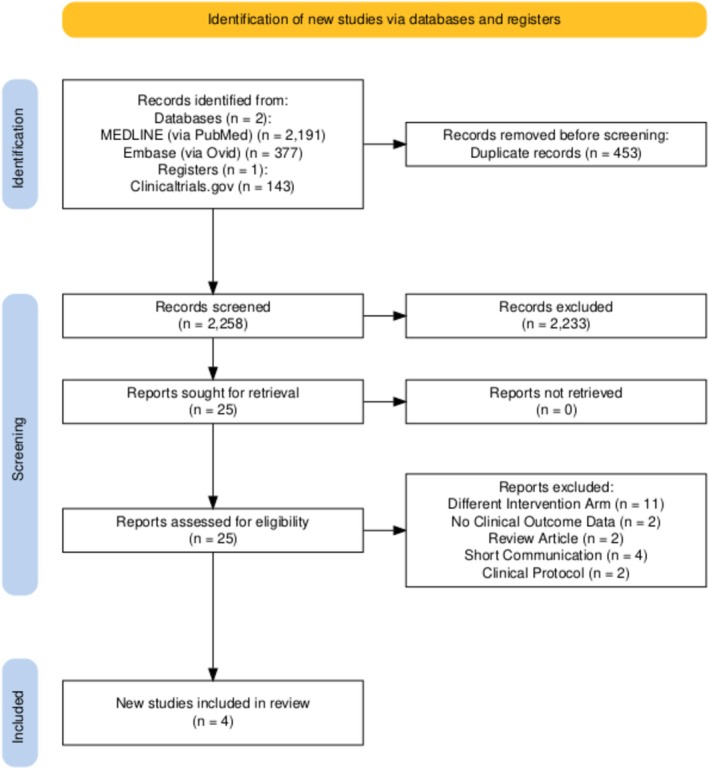
PRISMA flowchart.

The PRISMA search flow diagram is shown in Figure 1. The initial electronic database search yielded 2711 studies. After removal of duplicates, 2258 studies remained. Of these, 2233 were excluded during title and abstract screening because they did not meet the inclusion criteria. After thorough full‐text review of the remaining studies, 4 were ultimately eligible for inclusion in this systematic review and meta‐analysis [21, 22, 23, 24].

All the included studies were RCTs, including a total of 3333 participants. Of these, 1228 underwent CADe+EAC, while the control groups comprised 554 participants who underwent standard colonoscopy and 1236 who underwent CADe alone. The mean age (Standard Deviation) of participants in the CADe+EAC group was 61.59 (8.96) years, while that in the standard colonoscopy and CADe alone was 63.3 (8.76) and 62.68 (9.59) years respectively. The percentage of the male population across the studies was 44.7%. The mean Boston bowel preparation score was 7.4 (1.4) in the CADe+EAC group as compared to 7.79 (1.55) and 7.35 (1.45) in the standard colonoscopy and CADe alone groups respectively. Table 1 provides detailed characteristics of the included studies and patient demographics.

**TABLE 1:** Baseline characteristics of studies included in systematic review and meta‐analysis.

Study		Liu et al. 2024	Caillo et al. 2024	Spadaccini et al. 2023	Aniwan et al. 2023
Country		China	France	Italy and Switzerland	Thailand
Study type		Single‐centre study	Single‐centre study	Multi‐centre study	Single‐centre study
Study design		RCT	RCT	RCT	RCT
Intervention (I)		CADe+EAC	CADe+EAC	CADe+EAC	CADe+EAC
Control (C)		Standard colonoscopy and CADe alone	Standard colonoscopy and CADe alone	CADe alone	Standard colonoscopy and CADe alone
No. of participants (*n*)		1316	90	682	1245
Mean age in years (SD)	I	61.5 (9.9)	62 (11)	61.5 (9.9)	61.7 (7.00)
	C	61.30 (10.03)	61.9 (10.21)	61.30 (10.03)	62.3 (6.85)
Male sex (%)		44.76%	44.44%	52.35%	40.40%
Mean BBPS score (SD)	I	7.3 (1.7)	8.3 (0.9)	7.14 (1.32)	7.9 (1.26)
	C	7 (1.8)	8.25 (0.96)	7.13 (1.31)	8.13 (1.24)
Procedure settings		Hospital	Endoscopy centre	Endoscopy centres	Hospital
Computer‐aided detection system		OIP‐1 AI system	GI Genius (v2.0.1, Medtronic)	GI Genius (Medtronic)	CAD EYE
Endoscopist experience		50% experienced endoscopist with > 7 years experience	76.7% endoscopists > 1000 colonoscopies	All experienced endoscopist (> 500 colonoscopies/year)	Not specified
Indication		Screening (18%), Surveillance (28.2%), Diagnostic (53.7%)	FIT+ (30%), Personal/family history of adenoma (38%), Family history of cancer (26%), GI symptoms (5%)	Diagnostic (33.1%), FIT+ (21.6%), Surveillance (22.5%), Primary screening (25.2%)	Present family history of colorectal cancer (12.53%), Fecal occult blood positive (10.92%)

Abbreviations: BBPS, Boston bowel preparation scale; CADe, computer‐aided detection; EAC, EndoCuff vision assisted colonoscopy; RCT, randomized controlled trial.

### 3.2 Results of Meta‐Analysis

A detailed summary of the pooled clinical outcomes is provided in Table 2.

**TABLE 2:** Detailed summary of the pooled clinical outcomes.

Outcome	Pooled effect size (95% CI)	*p*	*I* ^2^
CADe+EAC vs. standard colonoscopy			
Adenoma detection rate	1.36 (1.19 to 1.56)	< 0.01	9%
Advanced adenoma detection rate	1.62 (1.16 to 2.25)	< 0.01	0%
Sessile serrated lesion detection rate	1.95 (1.46 to 2.61)	< 0.01	0%
Mean adenomas per colonoscopy	0.50 (0.49 to 0.52)	0	9%
Mean withdrawal time	−0.29 (−0.79 to 1.370)	0.60	68%
Mean insertion time	−0.64 (−1.61 to 0.33)	0.19	80%
CADe+EAC vs. CADe Alone			
Adenoma detection rate	1.13 (1.04 to 1.22)	< 0.01	0%
Advanced adenoma detection rate	1.21 (0.93 to 1.57)	0.16	21%
Sessile serrated lesion detection rate	1.16 (0.96 to 1.41)	0.1245	0%
Mean adenomas per colonoscopy	0.15 (0.07 to 0.24)	< 0.01	85%
Mean withdrawal time	−0.82 (−1.40 to −0.24)	< 0.01	0%
Mean insertion time	−0.88 (−1.54 to −0.22)	< 0.01	71%

Abbreviations: CADe, computer‐aided detection system; CI, confidence intervals; EAC, EndoCuff vision‐assisted colonoscopy; *I* ^2^, heterogeneity.

#### 3.2.1 ADR

**FIGURE 2:**
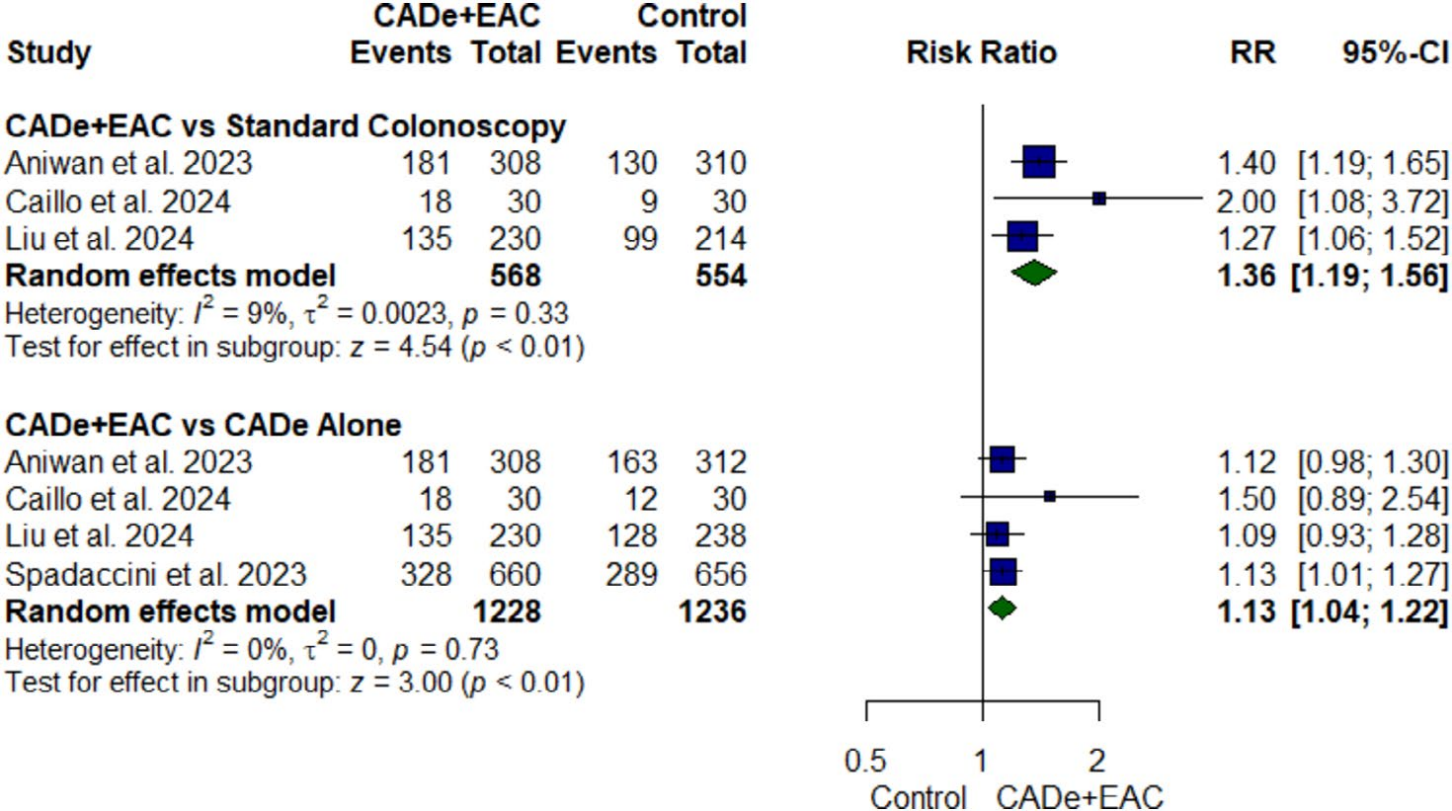
Forest plot for adenoma detection rate.

A total of four RCTs [21, 22, 23, 24], including 3018 participants demonstrated that CADe+EAC resulted in a significant 36% (RR: 1.36; 95% CI, 1.19 to 1.56; *p* value < 0.01, *I* ^2^ = 9%, Figure 2) and 13% (RR: 1.13; 95% CI, 1.04 to 1.22; *p* value < 0.01, *I* ^2^ = 0%, Figure 2) relative increase in ADR as compared to standard colonoscopy and CADe alone, respectively (Figure 2).

#### 3.2.2 AADR

**FIGURE 3:**
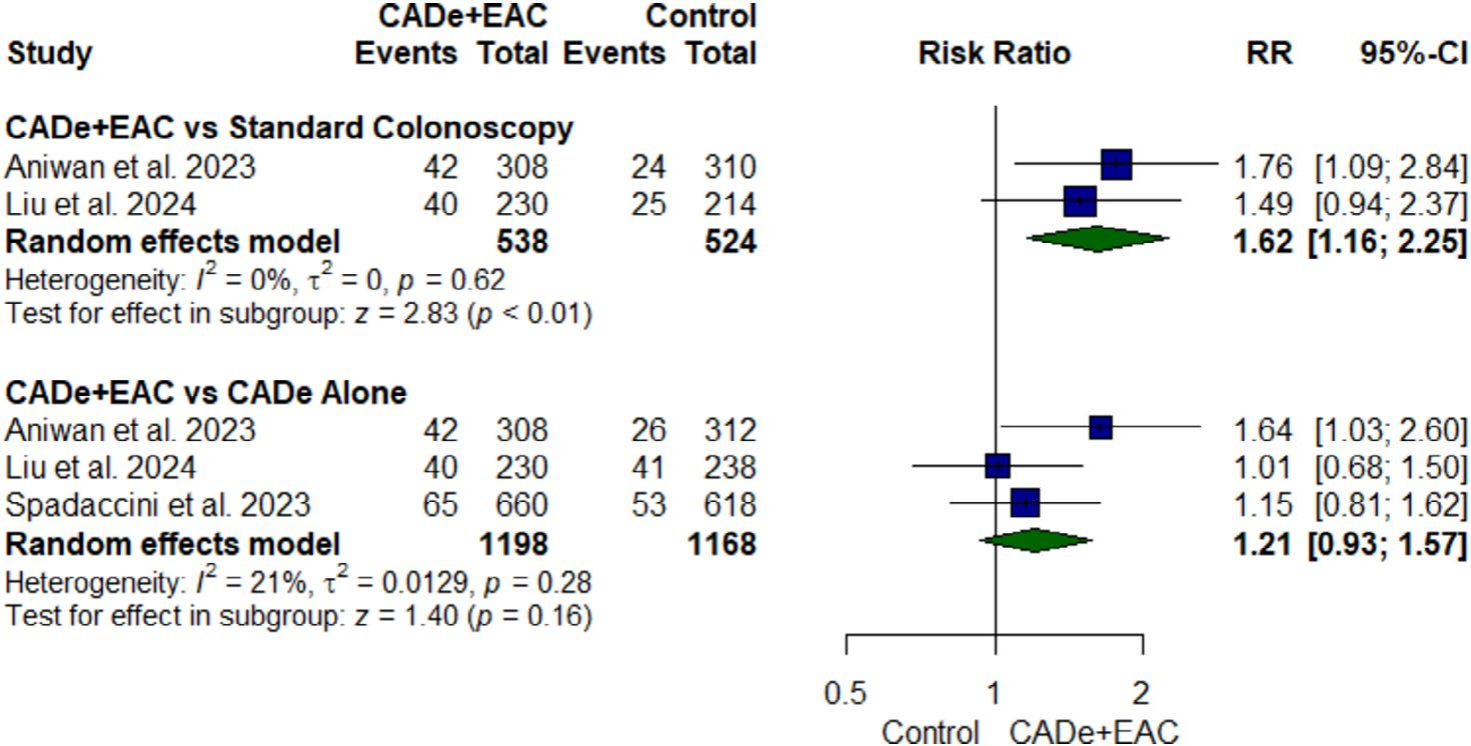
Forest plot for advanced adenoma detection rate.

For AADR, three RCTs [21, 23, 24] comprising 2890 participants were pooled (Figure 3). CADe+EAC group demonstrated a significant 62% increase in AADR as compared to standard colonoscopy (RR: 1.62; 95% CI, 1.16 to 2.25; *p* value < 0.01, *I* ^2^ = 0%, Figure 3). However, there was no statistically significant difference between CADe alone group and CADe+EAC group (RR: 1.21; 95% CI, 0.93 to 1.57; *p* value = 0.16, *I* ^2^ = 21%, Figure 3).

#### 3.2.3 SSLDR

**FIGURE 4:**
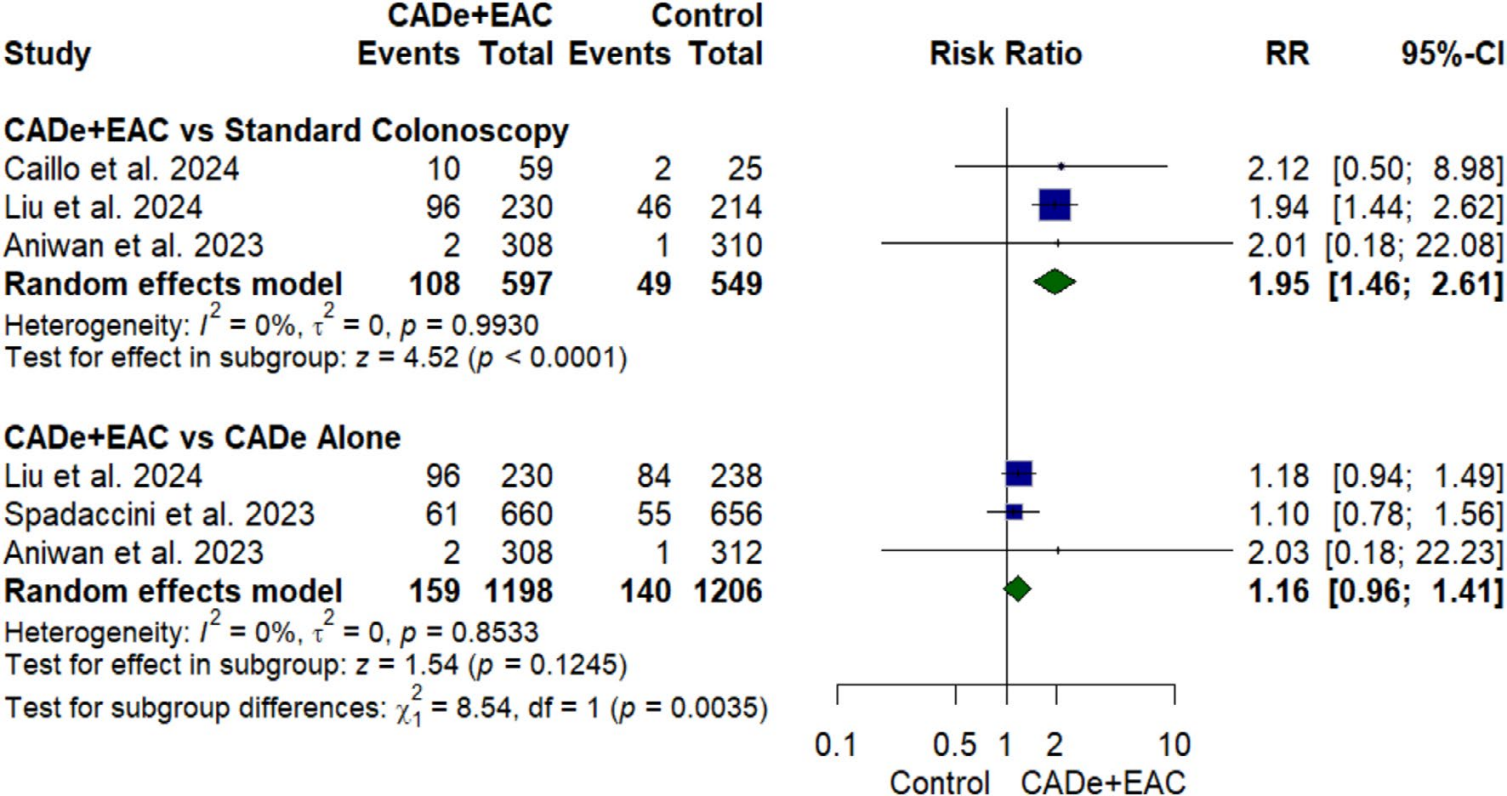
Forest plot for sessile serrated lesion detection rate.

Three RCTs [22, 23, 24] with 3012 participants were pooled for analysis of SSLDR (Figure 4). SSLDR was significantly higher in the CADe+EAC group with an increase of 95% as compared to standard colonoscopy (RR: 1.95; 95% CI, 1.46 to 2.61; *p* value < 0.01, *I* ^2^ = 0%, Figure 4). However, the results were comparable with the CADe alone group (RR: 1.16; 95% CI, 0.96 to 1.41; *p* = 0.1245, *I* ^2^ = 0%, Figure 4).

#### 3.2.4 Mean Adenomas Per Colonoscopy

**FIGURE 5:**
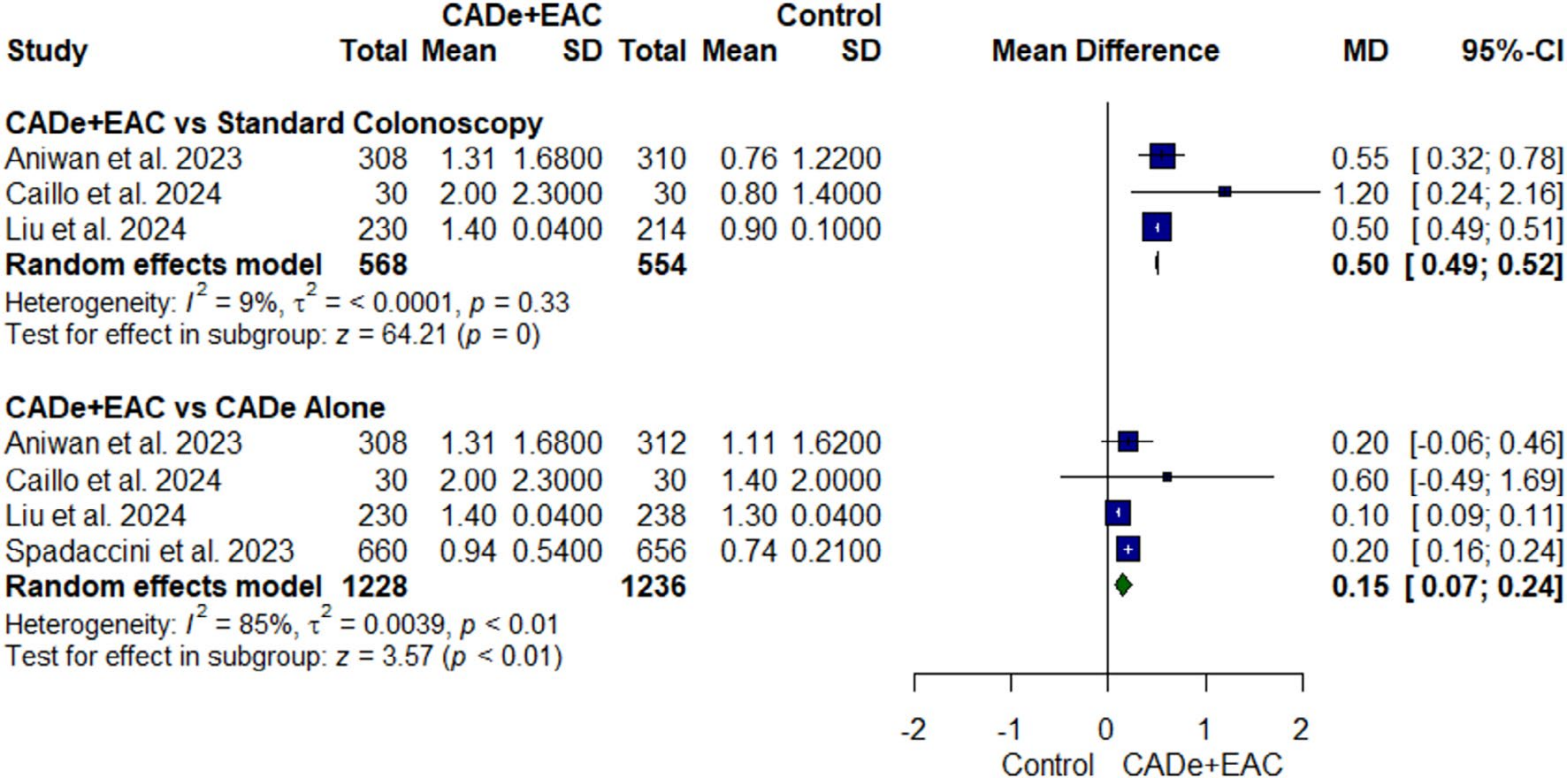
Forest plot for mean adenomas per colonoscopy.

Four RCTs [21, 22, 23, 24] with 3018 participants were pooled to analyze mean APC outcome (Figure 5). Mean APC was significantly higher in the CADe+EAC group as compared to CADe alone (MD: 0.15; 95% CI, 0.07 to 0.24; *p* value < 0.01, *I* ^2^ = 85%, Figure 5) and standard colonoscopy groups (MD: 0.50; 95% CI, 0.49 to 0.52; *p* value = 0, *I* ^2^ = 9%, Figure 5). In the leave‐one‐out analysis, the study by Liu et al. was identified as the primary source of heterogeneity in CADe+EAC versus CADe alone analysis. The exclusion of this study did not significantly affect the effect estimate of mean APC (MD: 0.20; 95% CI, 0.16 to 0.24; *p* < 0.01), but it eliminated heterogeneity (*I* ^2^ = 0%) (Figure S1).

#### 3.2.5 Mean Withdrawal Time

**FIGURE 6:**
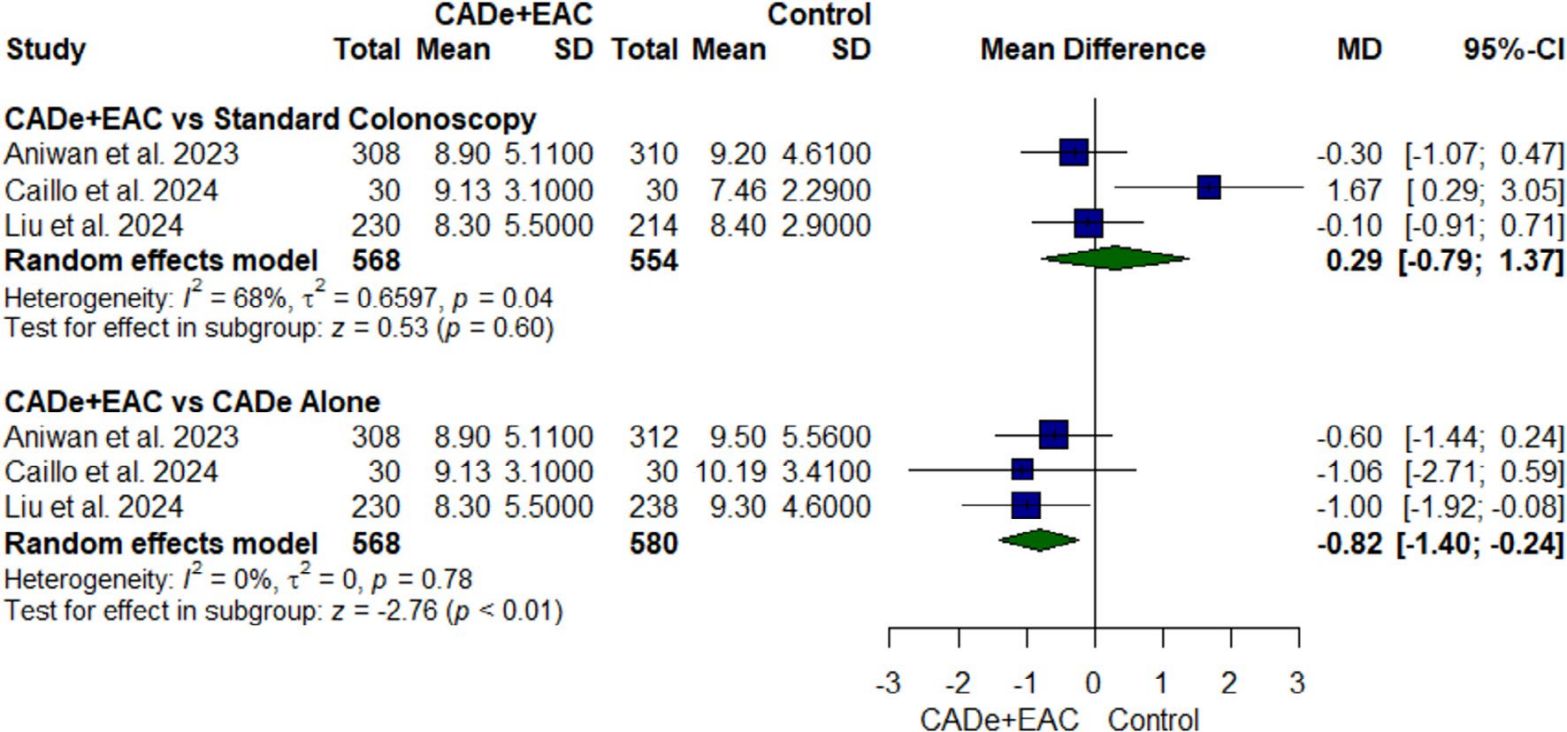
Forest plot for mean withdrawal time.

Three RCTs [21, 22, 23] were pooled for mean withdrawal time comprising 1702 participants (Figure 6). Mean withdrawal time was significantly lower in the CADe+EAC group as compared to CADe alone (MD: −0.82; 95% CI, −1.40 to −0.24; *p* value < 0.01, *I* ^2^ = 0%, Figure 6). However, the results were comparable with the standard colonoscopy group (MD: 0.29; 95% CI, −0.79 to 1.37; *p* value = 0.60, *I* ^2^ = 68%, Figure 6). In the leave‐one‐out analysis, the study by Caillo et al. was identified as the primary source of heterogeneity in CADe+EAC versus standard colonoscopy analysis. The exclusion of this study did not significantly affect the effect estimate of mean withdrawal time (MD: −0.82; 95% CI, −1.40 to −0.24; *p* < 0.01), but it eliminated heterogeneity (*I* ^2^ = 0%) (Figure S2).

#### 3.2.6 Mean Insertion Time

**FIGURE 7:**
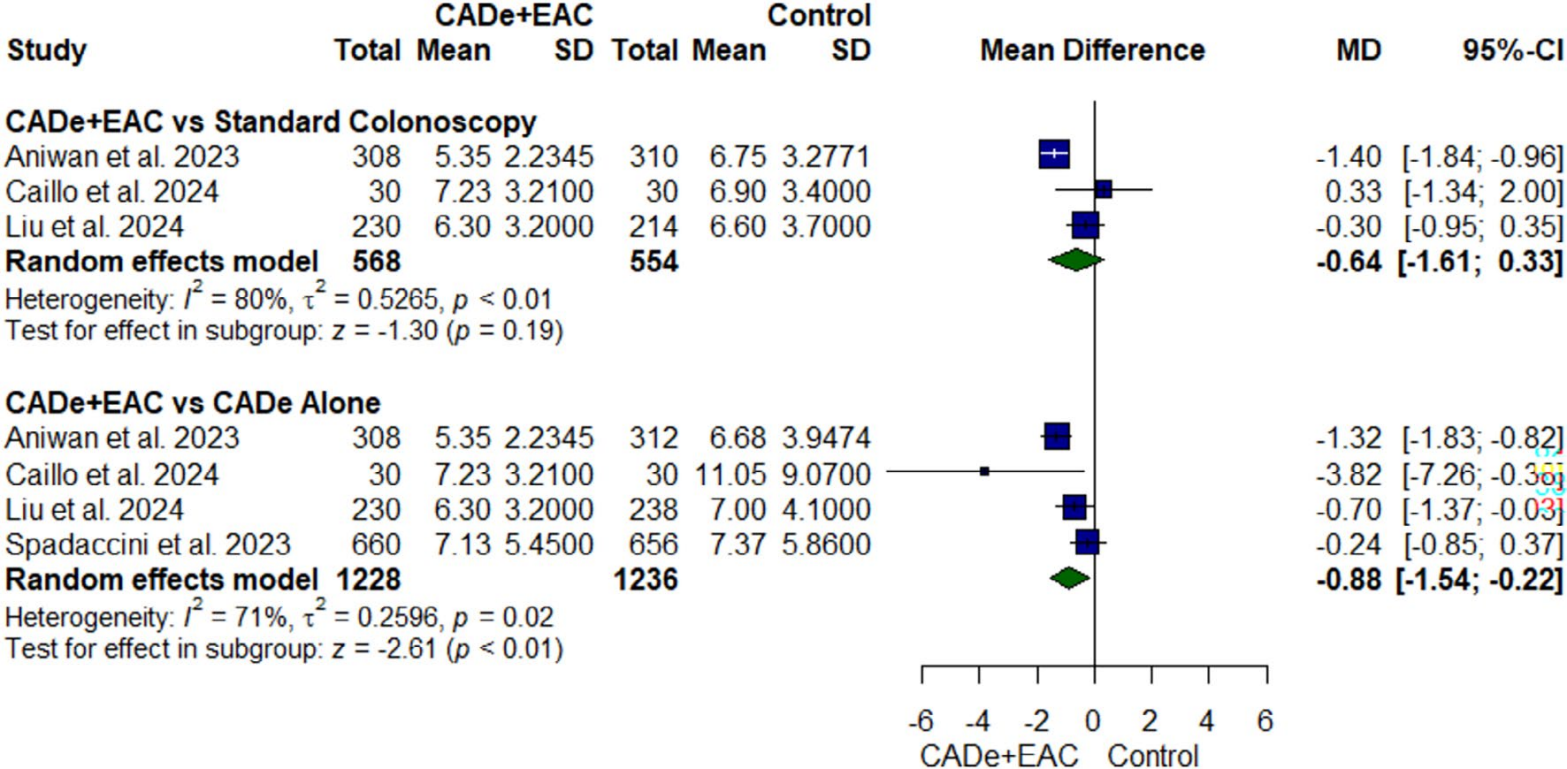
Forest plot for mean insertion time.

Four RCTs [21, 22, 23, 24] having 1122 participants were pooled for mean insertion time (Figure 7). Mean insertion time was significantly lower in the CADe+EAC group as compared to CADe alone (MD: −0.88; 95% CI, −1.54 to −0.22; *p* value < 0.01, *I* ^2^ = 71%, Figure 7). However, the results were comparable with the standard colonoscopy group (MD: −0.64; 95% CI, −1.61 to 0.33; *p* value = 0.19, *I* ^2^ = 80%, Figure 7). In the leave‐one‐out analysis, the study by Aniwan et al. was identified as the primary source of heterogeneity in CADe+EAC versus standard colonoscopy analysis. The exclusion of this study did not significantly affect the effect estimate of mean insertion time (MD: −0.22; 95% CI, −0.82 to 0.38; *p* = 0.48), but it eliminated heterogeneity (*I* ^2^ = 0%) (Figure S3).

### 3.3 Complications

Table 3 provides a summary of all reported complications from the included studies.

**TABLE 3:** Complications reported by studies included in the systematic review and meta‐analysis.

Study	CADe+EAC (n)	CADe alone (*n*)	Standard colonoscopy (n)
Liu et al. 2024	Incomplete colonoscopy (5)	Incomplete colonoscopy (5)	Incomplete colonoscopy (7)
Caillo et al. 2024	Postresection bleedings (7), Incomplete colonoscopy (1), No complications (22)	Postresection bleedings (2), Incomplete colonoscopy (1), No complications (27)	Postresection bleedings (2), Incomplete colonoscopy (0), No complications (28)
Spadaccini et al. 2023	N/R	N/R	N/R
Aniwan et al. 2023	Zero adverse events	Zero adverse events	Zero adverse events

Abbreviations: *n*, number of participants; N/R, not reported.

### 3.4 Quality Assessment

The results of the risk of bias assessment using the Cochrane Risk of Bias tool are shown in Figures S4 and S5. All the four clinical trials showed a low risk of bias in all five domains (Table 4).

**TABLE 4:** GRADE assessment of certainty of evidence.

Certainty assessment							No. of patients		Effect		Certainty
No. of studies	Study design	Risk of bias	Inconsistency	Indirectness	Imprecision	Other considerations	CADe+EAC	Standard or CADe alone	Relative (95% CI)	Absolute (95% CI)	
Advanced adenoma detection rate (Control = standard colonoscopy)											
2	Randomized trials	Not serious	Not serious	Not serious	Serious^f^	None	82/538 (15.2%)	49/524 (9.4%)	**RR 1.62** (1.16 to 2.25)	**58 more per 1000** (from 15 more to 117 more)	⨁⨁⨁◯Moderate^f^
Advanced adenoma detection rate (Control = CADe alone)											
3	Randomized trials	Not serious	Serious^c^	Not serious	Serious^b^	None	147/1198 (12.3%)	120/1168 (10.3%)	**RR 1.21** (0.93 to 1.57)	**22 more per 1000** (from 7 fewer to 59 more)	⨁⨁◯◯Low^b^, ^c^
Adenoma detection rate (Control = standard colonoscopy)											
3	randomized trials	Not serious	Serious^c^	Serious^a^	Serious^f^	None	334/568 (58.8%)	238/554 (43.0%)	**RR 1.36** (1.19 to 1.56)	**155 more per 1000** (from 82 more to 241 more)	⨁◯◯◯Very low^a^, ^c^, ^f^
Adenoma detection rate (Control = CADe alone)											
4	Randomized trials	Not serious	Serious^c^	Serious^a^	Not serious	None	662/1228 (53.9%)	592/1236 (47.9%)	**RR 1.13** (1.04 to 1.22)	**62 more per 1000** (from 19 more to 105 more)	⨁⨁◯◯Low^a^, ^c^
Insertion time (Control = standard colonoscopy)											
3	Randomized trials	Not serious	Very serious^d^	Serious^a^	Serious^b^	None			—	MD **0.64 lower** (1.61 lower to 0.33 higher)	⨁◯◯◯Very low^a^, ^b^, ^d^
Insertion time (Control = CADe alone)											
4	Randomized trials	Not serious	Very serious^e^	Serious^a^	Serious^f^	None			—	MD **0.88 lower** (1.54 lower to 0.22 lower)	⨁◯◯◯Very low^a^, ^e^, ^f^
Mean adenoma (Control = standard colonoscopy)											
3	randomized trials	Not serious	Serious^c^	Serious^a^	Not serious	None			—	MD **0.5 higher** (0.49 higher to 0.52 higher)	⨁⨁◯◯Low^a^, ^c^
Mean adenoma (Control = CADe alone)											
4	Randomized trials	Not serious	Very serious^e^	Serious^a^	Serious^f^	None			—	MD **0.15 higher** (0.07 higher to 0.24 higher)	⨁◯◯◯Very low^a^, ^e^, ^f^
Sessile serrated lesion detection rate (Control = standard colonoscopy)											
2	Randomized trials	Not serious	Not serious	Serious^a^	Not serious	None	106/289 (36.7%)	48/239 (20.1%)	**RR 1.95** (1.46 to 2.61)	**191 more per 1000** (from 92 more to 323 more)	⨁⨁⨁◯Moderate^a^
Sessile serrated lesion detection rate (Control = CADe alone)											
3	Randomized trials	Not serious	Serious^c^	Serious^a^	Serious^f^	None	167/949 (17.6%)	140/932 (15.0%)	**RR 1.37** (0.66 to 2.84)	**56 more per 1000** (from 51 fewer to 276 more)	⨁◯◯◯Very low^a^, ^c^, ^f^
Withdrawal time (Control = standard colonoscopy)											
3	Randomized trials	Not serious	Serious^e^	Serious^a^	Serious^f^	None			—	MD **0.29 higher** (0.79 lower to 1.37 higher)	⨁◯◯◯Very low^a^, ^e^, ^f^
Withdrawal time (Control = CADe alone)											
3	Randomized trials	Not serious	Not serious	Serious^a^	Serious^f^	None			—	MD **0.82 lower** (1.4 lower to 0.24 lower)	⨁⨁◯◯Low^a^, ^f^

Abbreviations: CI: confidence interval; MD: mean difference; RR: risk ratio.

^a^ Population age variations.

^b^ 95% CI includes null effect.

^c^ 95% CIs do not overlap.

^d^ Significant heterogeneity.

^e^ Significant heterogeneity; some 95% confidence intervals do not overlap.

^f^ 95% confidence interval includes appreciable benefit or harm.

## 4 Discussion

Our comprehensive systematic review and meta‐analysis of 4 RCTs including 3333 patients evaluated the outcomes of CADe combined with EAC versus CADe alone and standard colonoscopy. Our pooled analysis demonstrated a significant association of improved ADR, AADR, mean APC, and SSLDR in the CADe+EAC group compared to standard colonoscopy. No significant differences were observed in mean withdrawal time and mean insertion time between the CADe+EAC group and standard colonoscopy. When compared to CADe alone, a significant improvement in ADR and mean APC was reported in the CADe+EAC group. Additionally, the CADe+EAC group was observed to have statistically significant lower mean withdrawal time and mean insertion time. No statistically significant difference was seen for AADR and SSLDR in the CADe+EAC group. All the included studies had a low or moderate risk of bias.

Although colonoscopy is the gold standard for early CRC detection, it is limited by high adenoma miss rates [5, 25]. Recent interest has centered on the development of various methods and tools to enhance our capacity to inspect hard‐to‐see parts of the colon, such as behind folds and turns, in an attempt to improve the efficacy of colonoscopy [25]. Enhanced visualization devices and the incorporation of AI have been the epitome of recent gastrointestinal research [25, 26, 27, 28, 29, 30, 31, 32, 33, 34, 35]. We conducted the first systematic review and meta‐analysis that pooled the outcomes of EAC integrated with CADe to study the synergistic effect of these technologies.

Located on the tip of the scope, the Endocuff and its descendant Endocuff‐Vision are single‐use devices made up of a cylindrical core with one (Endocuff‐Vision) or two (Endocuff) rows of flexible projections [36]. A meta‐analysis conducted by Triantafyllou et al. [27] reported a significantly improved ADR in EAC compared to standard colonoscopy [RR = 1.18; 95% CI = 1.05–1.32]. Similar investigations conducted by Williet et al. [26] [RR = 1.20; 95% CI = 1.06–1.36] and Chin et al. [21] [Odds ratio (OR) = 1.49; 95% CI = 1.23–1.80] reported a significantly improved ADR in the EAC group compared to standard colonoscopy. Chin et al. [25] also reported improvement in SSLDR [OR = 2.34; 95% CI = 1.63–3.36]. The most recent meta‐analysis of 9140 patients by Walls et al. [29] reported that EAC significantly increased ADR [RR = 1.18; 95% CI = 1.09–1.29] and mean APC [MD = 0.19; 95% CI = 0.06–0.33] versus standard colonoscopy. These results are consistent with our findings. However, Williet et al. [26] and Triantafyllou et al. [27] did not report any significant improvements in AADR and mean APC in the EAC group compared to standard colonoscopy, which is in contrast with our results. Additionally, EAC has been found to be associated with reduced withdrawal and inspection times without decreasing lesion detection rates [37, 38]. This finding further supports our observation of significantly reduced insertion and withdrawal times with the combined use of CADe and EAC compared to CADe alone. However, this difference was not statistically significant when compared to standard colonoscopy. Similar results were reported in the COLODETECT 1 trial [22], one of the studies included in our analysis. In their findings, the particularly shorter insertion time observed in the standard colonoscopy group was primarily influenced by a single, unusually prolonged procedure in the CADe+EAC group [22]. This highlights how operator experience and specific procedural factors can substantially affect the overall outcomes.

Although research on CADe for colonoscopy started in the early 2000s [39, 40, 41], colonoscopists have been closely monitoring it ever since its sensitivity and specificity in real‐time analysis surpassed 90% [41, 42, 43]. Systematic review and meta‐analysis conducted by Hassan et al. in 2023 [26] [RR = 1.24; 95% CI = 1.16–1.33] and in 2021 [31] [RR = 1.44; 95% CI = 1.27–1.62], Soleymanjahi et al. [28] [rate ratio = 1.21; 95% CI = 1.15–1.28], Mohan et al. [29] [RR = 1.5; 95% CI = 1.3–1.72], and Spadaccini et al. [30] [OR = 1.78; 95% CI = 1.44–2.18] demonstrated a statistically significant improvement in pooled ADR in CADe groups when compared with standard colonoscopy. Soleymanjahi et al. [32] [MD = 0.53; 95% CI = 0.30–0.77], and Mohan et al. [33] [MD = 0.38; 95% CI = 0.05–0.72] also reported significantly longer mean withdrawal time in the CADe group. Nevertheless, an investigation by Patel et al. [35] concluded that there were no significant differences in ADR [RR = 1.11; 95% CI = 0.97–1.28], mean APC [MD = 0.14; 95% CI = −0.04–0.32], and withdrawal time [MD = 0.8; 95% CI = −0.18–0.90] in CADe assisted and standard colonoscopies. Therefore, with reported adenoma miss rates ranging from 13% to 20%, CADe‐assisted colonoscopies remain controversial [44]. However, a mucosal exposure device such as EAC can overcome the limitations of CADe by enhancing the exposure of the mucosal surface to CADe [21, 22]. The RCT conducted by Aniwan et al. [21] reported that integrating EAC and CADe resulted in a two‐fold increase in AADR compared to the control group [21]. Lui et al. [23] reported that the addition of Endocuff to AI resulted in a 4.9% increase in ADR.

CADe is an operator‐dependent tool designed not to replace endoscopists but to function as an adjunct that supports and enhances their diagnostic capabilities [45]. According to Spadaccini et al., a key advantage of CADe is its potential to democratize endoscopic practice by reducing the variability between novice and expert performance. In doing so, CADe may help elevate the overall standard of care, particularly in settings where endoscopist expertise varies [45]. This potential has been highlighted by several studies. Aniwan et al. demonstrated that CADe‐assisted colonoscopy consistently showed superior outcome results than standard colonoscopy, irrespective of the operator's experience level [21]. Lui et al. also reported no significant effect of CADe on individual endoscopists' performance based on generalized estimating equation analysis, suggesting that the tool contributes uniformly across varying levels of expertise [23]. Similarly, several studies demonstrated that although EAC improves the colonoscopy outcomes, it may not make a big difference for highly experienced endoscopists [46, 47]. On the contrary, a study conducted by Lee et al. [48] demonstrated that both the nurse and fellow groups exhibited limited performance compared to the experts when using the CADe system, particularly in detecting sessile serrated lesions and other subtle or challenging polyps. They concluded that although the CADe enhances the detection rates overall, its effectiveness depends on the knowledge and experience of the user, especially when it comes to difficult‐to‐detect lesions [48]. They also suggested the importance of providing trainees with more focused and comprehensive education, particularly in recognizing subtle and complex lesions [48].

Our study has some strengths and limitations. The main strength of our study is that, despite well‐established and continuously growing literature on this topic, we are the first to compare the combination of CADe+EAC versus CADe alone and standard colonoscopy. Additionally, all our included studies had a low or moderate risk of bias. Furthermore, we included RCTs in our study and performed the leave‐one‐out sensitivity analysis to study the individual effects of each included study, followed by a critical appraisal of our findings according to the GRADE criteria, indicating our rigorous and robust methodology. Additionally, although our analysis included a substantial sample size, we could include only 4 RCTs that matched our inclusion criteria, 3 of which were single‐centered. This highlights the need for further multi‐centered trials in diverse populations. Furthermore, the majority of our outcomes demonstrated low heterogeneity, likely due to comparable study and patient characteristics, including study design, mean age, mean BBPS scores, male‐to‐female ratio, and the involvement of experienced endoscopists performing the procedures. Only a few of our outcomes were subjected to significant statistical heterogeneity, although leave‐one‐out sensitivity analyses were conducted to address this issue. Variations in results may be attributed to differing indications for colonoscopy across the included studies, such as familial history, screening, surveillance, and diagnostic purposes. Additionally, different AI models were employed in all the included studies. It should be noted that the integration of CADe and EAC demonstrated better colonoscopy results, mostly for diminutive and proximal lesions, but not for large lesions [21, 24]. The necessity for these technologies is justified by the greater miss rates for proximal and diminutive lesions [24], but future investigations should be done on these combination technologies that help improve outcomes for large lesions as well. Moreover, the quantitative evaluation in the form of a pooled analysis for adverse events or complication rates was not possible since this data was not reported in the included studies.

## 5 Conclusion

Our findings highlight the synergistic potential of integrating EAC and CADe to optimize colonoscopy effectiveness by increasing detection outcomes. EAC+CADe showed improved outcomes against both CADe alone and standard colonoscopy. Nevertheless, to determine the precise effectiveness and success profile of this intervention, more extensive multi‐centered trials utilizing a variety of AI models and their subgroup analysis are needed. For optimal patient benefit, cost‐effective evaluations of these technologies should also be carried out and compared with alternative low‐cost methods.

## Funding

The authors have nothing to report.

## Conflicts of Interest

The authors declare no conflicts of interest.

## Supporting information


**Table S1:** Detailed search strategy of each included database.
**Figure S1:** Leave‐one‐out analysis for Mean Adenomas per Colonoscopy.
**Figure S2:** Leave‐one‐out analysis for Withdrawal time.
**Figure S3:** Leave‐one‐out analysis for Mean Insertion time.
**Figure S4:** Traffic plot of Risk of Bias Assessment.
**Figure S5:** Summary of Risk of Bias Assessment.

## Data Availability

The authors confirm that the data supporting the findings of this study are available within the article and its Supporting Information.
