# Supplementary material for: Integration of Endocuff‐Assisted and Computer‐Aided Colonoscopy: A Meta‐Analysis of Randomized Controlled Trials
Source: JGH Open. 2026 Feb 14;10(2):e70356. doi: 10.1002/jgh3.70356 (PMC12906277; doi:10.1002/jgh3.70356)

**Supplementary File**

**Integration of Endocuff-Assisted and Computer-Aided Colonoscopy: A Meta-Analysis of Randomized Controlled Trials**

This file is created by the authors to provide a better understanding of their work.

**Supplementary Table S1:** Detailed search strategy of each included database

| **Database** | **Search Strategy** | **Results** |
| --- | --- | --- |
| MEDLINE (via PubMed) | (Colonoscopies OR Colonoscopic Surgical Procedures OR Colonoscopic Surgical Procedure OR Procedure, Colonoscopic Surgical OR Procedures, Colonoscopic Surgical OR Surgical Procedure, Colonoscopic OR Colonoscopic Surgery OR Colonoscopic Surgeries OR Surgeries, Colonoscopic OR Surgery, Colonoscopic OR Surgical Procedures, Colonoscopic) AND (Computer-Assisted Surgeries OR Surgeries, Computer-Assisted OR Surgery, Computer Assisted OR Computer-Aided Surgery OR Computer-Aided Surgeries OR Computer Aided Surgery OR Surgeries, Computer-Aided OR Surgery, Computer-Aided OR Computer-Assisted Surgery OR Computer Assisted Surgery OR Surgery, Image-Guided OR Image-Guided Surgeries OR Surgeries, Image-Guided OR Surgery, Image Guided OR Image-Guided Surgery OR Image Guided Surgery OR Surgical Navigation OR Navigation, Surgical OR Artificial Intelligence OR AI OR EndoCuff OR Mucosal Exposal Device OR EndoCuff Vision OR endocuff-assisted colonoscopy) | **2,191** |
| EMBASE (via Ovid) | **SET#1**  (Colonoscopies or Colonoscopic Surgical Procedures or Colonoscopic Surgical Procedure or Procedure, Colonoscopic Surgical or Procedures, Colonoscopic Surgical or Surgical Procedure, Colonoscopic or Colonoscopic Surgery or Colonoscopic Surgeries or Surgeries, Colonoscopic or Surgery, Colonoscopic or Surgical Procedures, Colonoscopic).mp. [mp=title, abstract, heading word, drug trade name, original title, device manufacturer, drug manufacturer, device trade name, keyword heading word, floating subheading word, candidate term word]  **SET#2**  (Computer-Assisted Surgeries or Surgeries, Computer-Assisted or Surgery, Computer Assisted or Computer-Aided Surgery or Computer-Aided Surgeries or Computer Aided Surgery or Surgeries, Computer-Aided or Surgery, Computer-Aided or Computer-Assisted Surgery or Computer Assisted Surgery or Surgery, Image-Guided or Image-Guided Surgeries or Surgeries, Image-Guided or Surgery, Image Guided or Image-Guided Surgery or Image Guided Surgery or Surgical Navigation or Navigation, Surgical or Artificial Intelligence or AI).mp. [mp=title, abstract, heading word, drug trade name, original title, device manufacturer, drug manufacturer, device trade name, keyword heading word, floating subheading word, candidate term word]  **SET#3**  (EndoCuff or Mucosal Exposal Device or EndoCuff Vision or endocuff-assisted colonoscopy).mp. [mp=title, abstract, heading word, drug trade name, original title, device manufacturer, drug manufacturer, device trade name, keyword heading word, floating subheading word, candidate term word]  **SET#4**  2 or 3  **SET#5**  1 and 4 | **377** |
| Clinicaltrials.gov |  | **143** |
| **Total** |  | **2711** |

**Supplementary Figure S1:** Leave-one-out analysis for Mean Adenomas per Colonoscopy


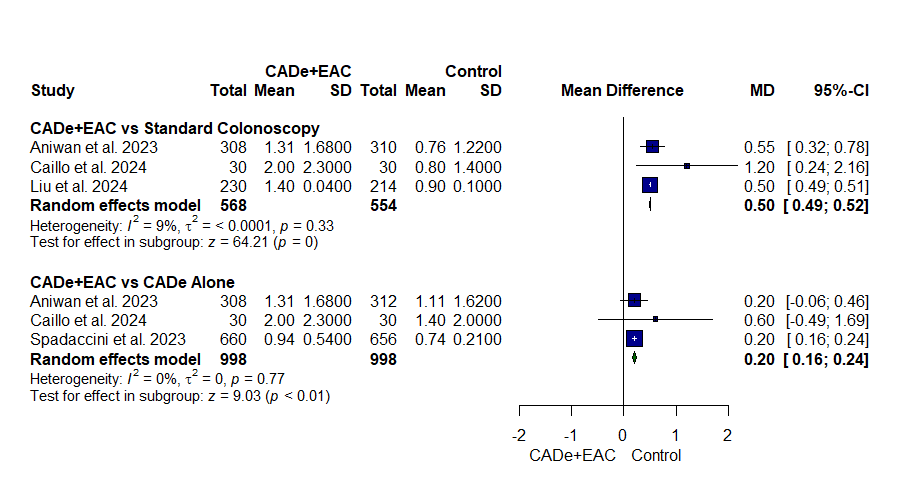


**Supplementary Figure S2:** Leave-one-out analysis for Withdrawal time


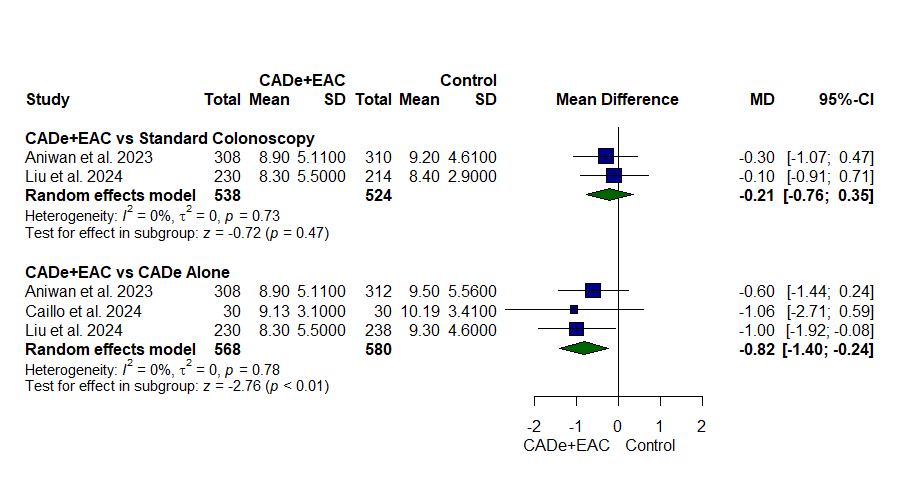


**Supplementary Figure S3:** Leave-one-out analysis for Mean Insertion time


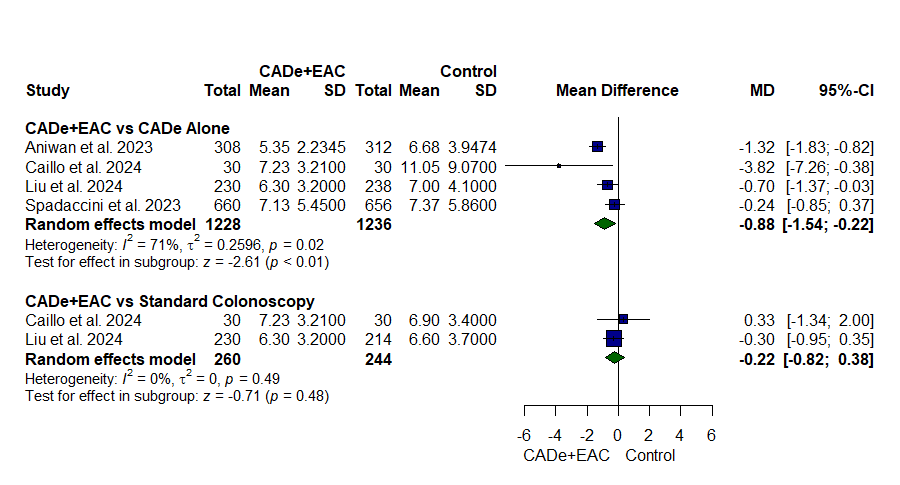


**Supplementary Figure S4:** Traffic plot of Risk of Bias Assessment


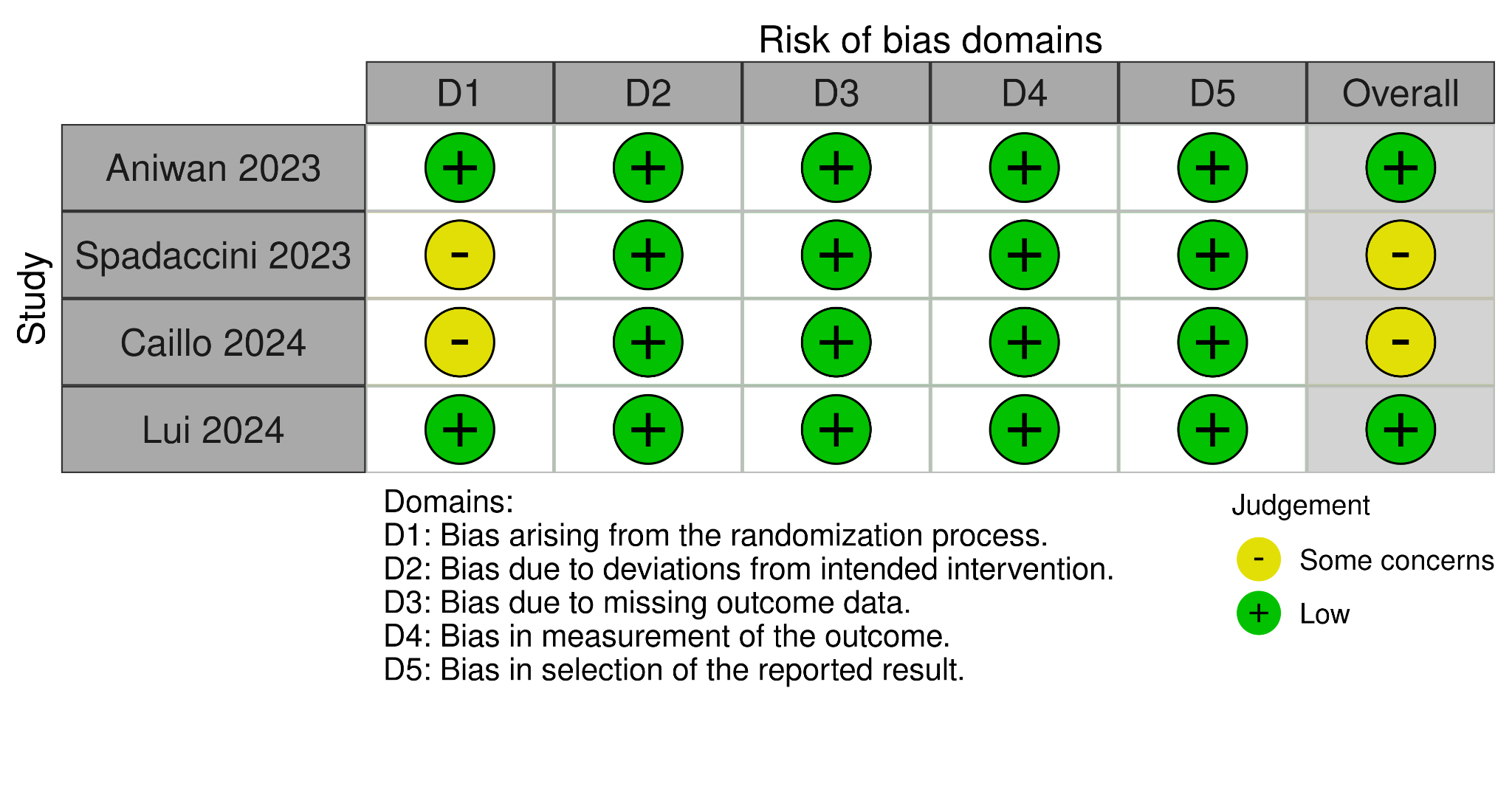


**Supplementary Figure S5:** Summary of Risk of Bias Assessment


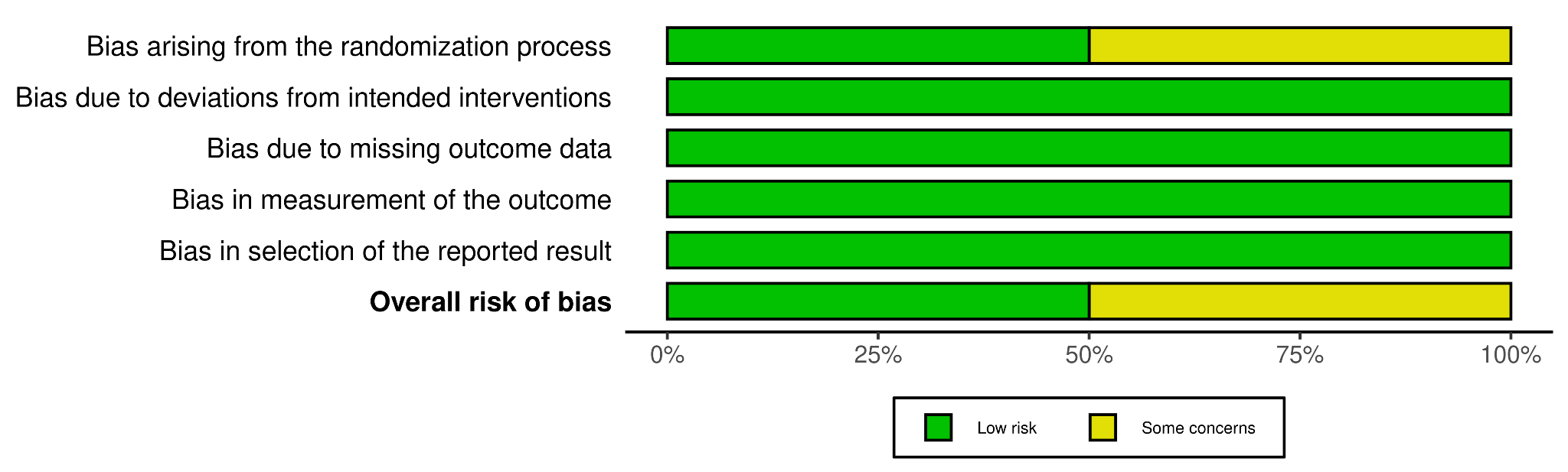

Supplement: Supplementary file 1 — Table S1: Detailed search strategy of each included database. Figure S1: Leave‐one‐out analysis for Mean Adenomas per Colonoscopy. Figure S2: Leave‐one‐out analysis for Withdrawal time. Figure S3: Leave‐one‐out analysis for Mean Insertion time. Figure S4: Traffic plot of Risk of Bias Assessment. Figure S5: Summary of Risk of Bias Assessment. [file JGH3-10-e70356-s001.docx]
